# Institutionalization of circular business models in the United States

**DOI:** 10.1111/jiec.70115

**Published:** 2025-11-08

**Authors:** Nancy Bocken, Matthew Coffay

**Affiliations:** 1 Maastricht Sustainability Institute, School of Business and Economics, Maastricht University, Maastricht, The Netherlands; 2 Centre for Sustainable Business, NHH Norwegian School of Economics, Bergen, Norway; 3 The International Institute for Industrial Environmental Economics (IIIEE), Lund University, Lund, Sweden; 4 Business School, University of Stavanger, Stavanger, Norway

**Keywords:** business models, circular economy, institutional theory, institutional work, North America, USA

## Abstract

The transition to a circular economy challenges existing regulatory frameworks, social norms, and belief systems: the dominant institutions. Moving from a linear to circular business model (CBM) is essential for sustainable business transformation. While CBMs have been widely studied in Europe, less is known about their emergence in the United States, where a national circular economy policy is still absent. This study explores how CBMs emerge in the United States, analyzing semi-structured interviews with companies and employing deductive thematic analysis alongside institutional work theory. First, we identify a variety of successful CBMs in the United States. Second, we present a framework of pathways for circular business innovation. Third, we present a “Circular Business Institutionalization Framework,” which bridges theories on institutional work and ecologies of business models. The frameworks offer insights into institutional leverage points and strategic positioning, with implications for guiding future sustainability transitions.

**Supplementary Information:**

The online version of this article (doi:10.1111/jiec.70115) contains supplementary material, which is available to authorized users.

## INTRODUCTION

Circular economy is often portrayed as a visionary concept for resolving global issues like climate change and resource scarcity (Geissdoerfer et al., 2017; Ghisellini et al., 2016) via resource preservation and nature restoration (Blomsma & Brennan, 2017) and enabling strategies like narrowing, slowing, and closing resource loops (Bocken et al., 2016; Konietzko et al., 2020). However, many companies’ business models are linear, and scaled circular business model (CBM) examples are rare. Still, the transition to circularity is essential for a broader sustainability transition.

Several CBM typologies exist (e.g., Henry et al., 2020; Lüdeke-Freund et al., 2019; Tukker, 2015) while organizations such as the Ellen MacArthur Foundation and Circle Economy publish circular economy cases. Moreover, CBM cases emerge in the literature (e.g., Vermunt et al., 2019). However, existing work comes with limitations. First, little is known about the institutional (i.e., regulative, cognitive, and normative; Geels, 2004; Scott, 2013) context in which CBMs develop (Van Gaubergen et al., 2023) and how their emergence depends on this context and co-existence with other business models (Boons & Bocken, 2018). Second, there is a lack of understanding of international manifestations of CBMs. Differences in institutional contexts like policies (Türkeli et al., 2018), norms, and values (Camacho-Otero et al., 2018; Corvellec et al., 2022) suggest that CBMs emerge differently. Research has started to develop an understanding of factors that drive CBM success but not yet with an institutional focus.

The emergence of CBMs needs further exploration as regional implementations differ greatly (Fraser et al., 2023). In this study, the US context is investigated. The United States is amongst the largest economies in the world, as well as the second largest polluter (Liu & Raftery, 2021; Pforzheimer & Truelove, 2021), generating 2.2 kg of waste per person per day (EPA, 2022a). Most of the waste (62%) discarded by homes and businesses is ultimately landfilled or incinerated (Pforzheimer & Truelove, 2021). Compared to other developed nations, US consumption and waste per capita are high, with relatively low recycling rates (Smith, 2019). While the United States has adopted practices such as remanufacturing and recycling (Parker et al., 2015) and industrial symbiosis (Chertow, 2000; Neves et al., 2019) and cities and states are starting to set examples, a national or regional circular economy framework like those in Europe or China is lacking (Ghisellini et al., 2016; Ren et al., 2024). This study investigates the following research question: *How do circular business models emerge in the United State*s*?*

This question is studied through the lenses of institutional work (Lawrence & Suddaby, 2006; Zvolska et al., 2019), and ecologies of business models—the notion that both circular and linear business models evolve, compete, and co-exist to varying extents alongside one another—to investigate how CBMs emerge compared to others (Bocken et al., 2019; Boons & Bocken, 2018). Our study focuses on the institutionalization of CBMs by analyzing how organizations have successfully implemented CBMs. This study illustrates how institutional work can advance the circular economy agenda by illuminating pathways and proposing a novel Circular Business Institutionalization Framework, feeding growing policy and business interests.

## BACKGROUND

### Institutional work and circular business models

CBMs are a new field of research (Ding et al., 2023; Lüdeke-Freund et al., 2019) following earlier research on sustainable business models (Boons & Lüdeke-Freund, 2013; Stubbs & Cocklin, 2008) and Product Service Systems (PSS) (Tukker, 2015). As a subset of sustainable business models, CBMs focus on resource loops and regeneration (Geissdoerfer et al., 2020; Konietzko et al., 2020). Examples include long-lasting design, value optimization (rental), value recovery (remanufacturing), and network models (Achterberg et al., 2016), aiming to retain and recover value as visualized in the value hill (c.f. Haase et al., 2024).

Studies on circular economy have often focused on Europe (Arranz & Arroyabe, 2023), and comparing regions like Europe to China with different policies (Ghissellini et al., 2016; Türkeli et al., 2018). Ambition levels differ with recycling models more omnipresent than service models like rental or sharing (Ritala et al., 2018). While several studies have examined emerging regulatory contexts like The Netherlands (Siderius & Poldner, 2021; Vermunt et al., 2019), France and Singapore (Rezvani Ghomi et al., 2021), and Europe (Briguglio et al., 2021), research on regions with “institutional voids” (e.g., lacking legislation) is lacking.

Thus far, institutional voids, where societal and environmental issues remain unaddressed, have created spaces for business model innovation by companies (Yunus et al., 2010). Despite lacking policies and normative expectations, new business models continue to emerge (Wells, 2018), potentially disrupting existing markets and competitive landscapes (Bower & Christensen, 1995). For example, researchers discussed new business models in energy and mobility as the basis for sustainability transitions (Bidmon & Knab, 2018; Sarasini & Linder, 2018). Yet, institutions may also reinforce issues such as poverty and resource extraction (Vellema et al., 2020). Hence new ways of organizing are needed to break through dominant institutions (Vellema et al., 2020). The business model has been investigated as an important part of the transition (Bidmon & Knab, 2018), like the move from a dominant unsustainable linear to a CBM. Studying CBMs contextually will help advance knowledge on how to nurture their development.

Research on institutional work (Graf & Jacobsen, 2021; Lawrence & Suddaby, 2006; Zvolksa et al., 2019) investigates how organizations might maintain, create, or disrupt institutions and is therefore useful to understand transitions. Some actors work to change institutions whereas others prefer to block change and maintain existing institutions (Graf & Jacobsen, 2021). There are three types of institutional work: (1) political work to change regulatory institutions; (2) reconfiguration of actors’ belief systems, to modify normative institutions; and (3) changing boundaries of meaning systems, by altering cognitive institutions (Zvolska et al., 2019, p. 669 based on Lawrence & Suddaby, 2006). Institutional work is useful to investigate ways in which organizations through innovative business models can break through norms and values, cultural-cognitive systems, and dominant rules and regulations (Lawrence & Suddaby, 2006). Institutional work reframes the way we think about institutions as more dynamic than static. While actors in an organization are to some extent constrained by institutional structures and norms, they also act on and actively shape those same institutional features (Phillips & Lawrence, 2012). Organizations might therefore either maintain, create, or disrupt institutions (Zvolksa et al., 2019). Examples of maintaining institutions might be oil companies perpetuating their extractive unsustainable models or energy companies relying on fossil fuels rather than pursuing a renewable energy model (Graf & Jacobsen, 2021).

To understand the emergence of CBMs, we draw on institutional *theory*, which offers a broad perspective on rules, norms, beliefs, and organizational activity (Scott, 2013). In so doing, we adopt an institutional *lens* in the sense that we analyze how these institutional theory elements influence CBM development. This allows us to illuminate CBM emergence in terms of institutional *work*, or how organizations create or disrupt institutions (Lawrence & Suddaby, 2006).

While recent work has applied institutional theory to highlight the role of consumption policy in supporting CBMs (Arranz & Arroyabe, 2023), little if any research exists which examines institutional *work* related to CBMs. Some studies investigated institutional work in advancing the circular economy and sustainability transitions more generally (Arranz et al., 2022; Buenen & Patterson, 2019; Giezen, 2018; Närvänen et al., 2021). In addition to its application for circular economy, institutional work could be a relevant research lens for CBM research specifically (Bocken & Shirahada, 2025).

### Ecologies of (circular) business models to understand their emergence

CBMs emerge in an “ecology” of business models (Bocken et al., 2019; Boons & Bocken, 2018) in which they co-exist and compete with dominant linear models. As an analogue to natural ecosystems (Boons, 2009; Tsujimoto et al., 2018) highlighting niche competition (Dijk et al., 2016; Geels, 2018), the “ecologies” concept is especially salient for the circular economy (Blomsma et al., 2023; Kanda et al., 2021). It is important to understand CBMs in these terms as their net impact can be hard to assess, shaped as it is by interactions with other models which contributes to overall resource flows. Therefore, we analyze the ecologies in which US CBMs emerge.

### Conceptual framework: Institutional work and “ecologies of business models”

While few studies investigate business sustainability in terms of institutional work (Bonomi et al., 2020; Giezen, 2018; Vellema et al., 2020; Zvolska et al., 2019), institutional work is important in legislative voids where actors seek to transform institutions (Powell & DiMaggio, 2012; Vellema et al., 2020). Here, we bridge the theory on institutional work (Zvolska et al., 2019) and ecologies of business models (Bocken et al., 2019; Boons & Bocken, 2018), because of complementaries between the theories (see Bocken & Shirahada, 2025). Both theories recognize the role of organizations in challenging the status quo and consider the impact of organizations on a larger system. Yet, institutional work has roots in institutional theory (Scott, 2013) whereas the “ecologies of business models” theory has origins in the work on ecology and evolving organizations (Aldrich, 1999) and natural ecosystems (Boons, 2009; Boons & Bocken, 2018). The ecologies of business models theory adds a complementary perspective to institutional work by supporting the understanding of how business models evolve vis-à-vis others. Bridging these theories can help illuminate pathways for businesses seeking to transform institutions within a given context or ecosystem.

This paper focuses only on the creation and disruption of institutions, as presented in Table 1, since CBM innovation by its nature challenges the linear economy (Fischer & Pascucci, 2017). Working deductively, we synthesize key concepts from institutional work and the ecologies of business models literatures (Table 1). From Lawrence and Suddaby (2006), we adopted the institutional work categories of “regulatory,” “normative,” and “cultural cognitive” which build on Scott (2013), and integrated the different strategies for each as summarized by Zvolska et al. (2019) (Table 1). From Boons and Bocken (2018) and Bocken et al. (2019), we incorporated the CBM strategy (modifying, creating, and disrupting) and the dependencies within an ecology of business models (mutualistic–symbiotic vs. competing–parasitic). Table 1 brings together these perspectives in a conceptual framework to support CBM implementation by business in different contexts. This framework is the starting point for our empirical study as presented next.

**TABLE 1:** Conceptual framework of circular business model implementation in different contexts. Based on: (A) Zvolska et al. (2019); (B) Lawrence & Suddaby (2006), (C) Boons & Bocken (2018), (D) Bocken et al. (2019).

Institutional work			
**Ecologies of business models**			
**Circular business model strategy (C, D)**	Modifying relations and dependencies (C, D) Disrupting the linear model (C, D) Creating an entirely new circular model (C, D)		
**Dependencies on others (weak or strong) (C, D)**	Mutualistic and symbiotic dependencies (C, D) Competing or parasitic dependencies (C, D)		
	**Regulatory** (A, B)	**Normative work** (A, B)	**Cultural-cognitive work** (A, B)
**Creating institutions** (A, B)	Lobbying and litigation, advocacy Delimiting organizational fields, for example, through membership and policies	Self-identification (connecting individual with organizational values) Changing traditional meanings Creating new norms Organizing for a unified voice, creative normative networks	Isomorphic mimicry, imitation Constructing new meaning systems Educating
**Disrupting institutions** (A, B)	Removing privileges, disconnect sanctions	Undermining moral grounds	Undermining assumptions and beliefs

## METHODS

### Research design and data selection

Compared with Europe and China, US CBM research and policy are limited, reflecting the absence of national legislation (Ghisellini et al., 2016). While cities engage in experimentation and some firms have implemented recycling and remanufacturing, strategies like slowing the loop and regeneration are nascent (Bloomberg, 2023; Bocken & Geradts, 2022; USITC, 2012). Extant barriers include regulatory gaps for battery recycling, low landfill costs which undermine circularity, and lagging remanufacturing policy (Awan & Sroufe, 2022; Heath et al., 2022; Hopkinson et al., 2018). Meanwhile, EU policy spillover (e.g., Digital Product Passports (DPP) and the Corporate Sustainability Reporting Directive, or, CSRD, will likely impact thousands of US firms in the coming years, highlighting the importance of generating new knowledge in this area (Deloitte, 2023; European Commission, 2023; WBCSD, 2023).

To study how CBMs emerge in the US context, we take an institutional work lens, complemented with an “ecologies of business models” perspective (Table 1). According to Lawrence et al. (2013), institutional work focuses on the “messy day-to-day practices.” Hence, we focus on the current work, unfolding in everyday practices and specific activities in creating and disrupting institutions (see Zvolska et al., 2019). We chose semi-structured interviews because of the need for more probing, open-ended questions, and the desire to know the independent thoughts of each individual in a group. It also gives space for “examining uncharted territory with unknown but potential momentous issues,” giving the interviewer maximum latitude to spot useful leads and pursue them (Adams, 2015, p. 493).

### Sample selection

Interviews took place between July and September 2023. The main inclusion criterion for the study is the successful implementation of a CBM innovation in some aspect of the company's operations, in a company context with active operations in the United States. Interviewees were contacted based on their pursuit of CBM innovation in the organization, using the first author's network and snowballing for further interviewees. The goal was to study institutional work across a spectrum of firm types and CBMs. Therefore, a heterogeneous sample of firm types and sectors was selected. Exploratory conversations or email exchanges were conducted before the interview to assess suitability for the in-depth interview. Data collection continued until saturation was reached and no novel mechanisms for CBM institutionalization were identified. Table 2 includes the final set of interviewees in chronological order.

**TABLE 2:** Interviewees.

#	Company	Industry	Firm size	Role	Interview date	Duration
1	IKEA	Consumer furniture	MNC	Country Sustainability Manager (USA)	24 July, 2023	47 min
2	Vitsœ	Consumer furniture	SME	Planner, Responsible New York Office	28 July, 2023	35 min (plus email exchanges before)
3	Philips	Healthcare company	MNC	(Former) Senior Product Designer	8 August, 2023	50 min
4				Senior Director Sustainable Development	31 August, 2023	1 h
5				Global Trade-In Lead, Circular Solutions	8 September, 2023	28 min
6	ZZ Driggs	Consumer and enterprise furniture	SME	Founder and CEO	8 August, 2023	59 min
7	Davies Office	Office furniture	SME	Vice President	11 August, 2023	56 min
8	Staples	Office supply retail	MNC	(Former) VP Sustainability	18 August, 2023	1 h, 11 min
9	Caterpillar	Construction, mining and other engineering equipment	MNC	Director Business Development, Remanufacturing Unit	29 August, 2023	37 min
10	Sunnking	Electronics recycling services	SME	President	22 August, 2023	1 hour, 10 min
11	Earthster	Environmental assessment services	SME	Co-Founder	1 September, 2023	54 min
12	Canon	Document and printing services	MNC	Senior Vice President Business Operations USA	5 September, 2023	35 min (plus 1-h exploratory conversation before)
13	Chanel	Luxury fashion house	MNC	Senior Group Director, Sustainability	6 September, 2023	30 min (plus 30-min exploratory conversation before)
14	SAP	Enterprise resource planning systems	MNC	Head of Product Management, Sustainability	8 September, 2023	25 min (plus follow-up emails)
15	Toyota	Mobility	MNC	Vice President and Senior Manager	22 September, 2023	45 min (plus 1-h exploratory conversation before)
16	Loop (Terracycle)	Reuse ecosystem for consumer products	SME	Chief Administrative Officer	22 September, 2023	46 min (plus 30-min exploratory conversation before)
17	Fernish	Furniture rental	SME	CEO	22 September, 2023	39 min
18	Dyson	Consumer electronics	MNC	Senior Manager Experience Design	25 September, 2023	28 min

Abbreviations: MNC, multinational corporation; SME, small-medium enterprise.

### Data collection

The interview guide was developed based on Table 1, bringing together the perspectives on institutional work (Zvolska et al., 2019) and ecologies of business models (Bocken et al., 2019; Boons & Bocken, 2018). The study participants were asked to explain the CBM in their own words, how it emerged in relation to other business models (ecologies of business models) and the institutional perspectives (normative, cultural cognitive, and regulatory) on the CBM innovation. The full interview guide can be found in Appendix A of Supporting Information S1. The first author conducted the interviews and transcribed the data starting with the automatic transcripts from the video conferencing tools (Zoom and MS Teams), which were corrected for language mistakes before starting the coding process.

### Data analysis

Using deductive thematic analysis (Braun & Clarke, 2006; Clarke et al., 2015), we began by analyzing themes in Table 1. The authors coded the data for CBM strategy, dependencies on others, and creative/disruptive institutional work (including normative, regulatory, and cultural cognitive). This resulted in the themes presented in Section 4, which were further refined through the authors’ discussions and validated by interviewees (which resulted in minor quote corrections).

### Limitations

Our method presents some limitations. First, the sample was relatively small. A much larger sample size could have been achieved with a different method—for example, by conducting a survey using a questionnaire. However, as our study was exploratory, we opted for semi-structured interviews to allow for the emergence of unanticipated themes and insights. Second, the sample was heterogeneous including a variety of businesses, some with a head office outside the United States due to the broad focus on company involvement in CBM innovation. The rationale was to include a wide variety of CBM examples, as the research area is still nascent, which led to a diversity of companies in our sample.

## RESULTS

The interview data revealed (1) a typology of nascent CBMs in the United States, (2) a dynamic ecology of emergent CBMs evolving alongside existing linear models in dependent, creative, and disruptive ways; (3) both symbiotic and parasitic dependencies between these emergent CBMs and the existing dominant linear models; (4) extensive creative (and limited disruptive) institutional work impacting norms, culture/cognition, and the regulatory landscape; and (5) pathways to CBM implementation in the US context. These results are summarized next and further discussed in Section 5.

### Emergence of circular business models in the United States

For clarity, we classified the types of CBMs pursued by the companies according to those in Achterberg et al. (2016): (1) Premium pricing and durability, (2) PSS like rental and lease, (3) Buy- or take-back and resale models, (4) Buy- or take-back and reprocessing models, and (5) Facilitator and assessment models (Table 3). We also checked these classifications with the companies to verify the types in Table 3. We further included the “value hill type” for each business model, for additional context.

**TABLE 3:** Typology of circular business model types encountered in the United States.

Value hill type	Circular business model type	Definition	Companies
Circular design (uphill)	Premium pricing and durability	Higher pricing for higher quality, longer-lasting products	Vitsœ Chanel
Optimal use (tophill)	Product service system (PSS), for example, rental, product as a service	Companies pursuing rental, lease, and service models to enable direct reuse supported by strategies like repair and refurbishment	ZZ Driggs Fernish Philips
Value recovery (downhill)	Buy- or take-back and resale	Buy back and resell used goods with minimal or no repair, or refurbishment processes	IKEA Philips Davies Office
Value recovery (downhill)	Buy- or take-back and remanufacturing, parts harvesting, or recycling	Own remanufacturing: OEMs taking back own products for remanufacturing, parts harvesting or recycling	Caterpillar Canon Dyson Philips Toyota
		Gap exploiter: Companies remanufacturing and recycling others’ (OEM) products	Sunnking Davies Office
		Platforms enabling remanufacturing	Loop Staples
Network organization (cross-hill)	Facilitator and assessor	Facilitator: Enabling circular business models, for example, through software solutions	SAP
		Assessor: Supporting the assessment of impact of circular business models	Earthster

*Note*: Types classified according to the value hill (Achterberg et al., 2016).

First, it should be noted that some companies (like Philips and Davies Office) pursue multiple business models. For example, at Davies Office, when a product is traded in, it is checked according to the “R-ladder” to decide whether the product can be remanufactured to an as good or better than new state, refurbished to good functional standards or even upgraded, or whether parts may be harvested. In the worst case, a product may be recycled.

Second, for several companies, like IKEA, Philips, and Dyson, the new CBM co-exists with a linear model pursued by the business. For others (Vitsœ, Loop, and Davies Office), the CBM as presented is the dominant one.

### Ecologies of circular versus linear business models

This section shows how companies position their business model vis-à-vis others: modifying, disrupting the linear model, or creating an entirely new model (see Appendix B, Table B1 of Supporting Information S1), and how these business models relate.

First, there is evidence of *modification*, either because incumbent companies in the same sector are already doing the same (e.g., selling long-lasting goods) so it is not seen as disruptive, or because companies do not see it as possible yet in the United States to disrupt with a fully novel circular model. We found evidence of mutualistic/symbiotic dependencies and competing/parasitic dependencies on others (see Appendix B, Table B2 of Supporting Information S1). A number of interviewees (1 and 14) described mutualistic dependencies, with the expectation that they would need to work with many stakeholders in order to achieve circular outcomes. Others (e.g., interviewee 18) noted logistics and vendor dependency challenges uniquely present in the United States such as the lack of an extensive rail system and the limited coverage provided by individual vendors, combined with conflicting regulatory environments in different states—all of which results in the need to “patchwork” a solution.

Second, *disruptions* originate from both incumbent businesses like IKEA (secondhand offering available across the United States), Davies Office (reused furniture), and Chanel (disruptions to the linear economy view) as well as newer organizations like Sunnking who describe themselves as “disruptive because we do (…) both recycling and resell.” Disruptions compete with incumbent business models but also internally. Disruptive models sometimes literally compete for space: interviewee 1 described internal dependencies between the existing linear business model and the competing CBM, with the latter dependent upon physical space and labor which would otherwise be allocated to the former. Even in disruptive models, there are not only competing, but also mutualistic dependencies (e.g., Davies Office relying on quality furniture by incumbents).

Third and finally, CBMs are *created* by newer companies (up to a decade old) like Fernish who created “circular logistics” for furniture and made this inherent to their business model. Such models typically co-exist with incumbent linear models. Interviewee (8) indicated the need to engage in pre-competitive cooperation with companies in the same or adjacent industries to normalize a newly created CBM.

### Creating and disrupting institutions?

Next to the positioning of the business model vis-à-vis others, we identified forms of institutional work in the regulatory, normative, and cultural-cognitive dimensions, mostly related to *creating* rather than disrupting institutions.

#### Creating institutions

In the regulatory context (Appendix B, Table B3 of Supporting Information S1), some interviewees (1, 10) were engaged in active lobbying and advocacy to modify policies or introduce new policies that could enable the circular economy. For example, some US state-level policy creates complications for value recovery-type buyback and resale business models, for example, buyback and resale of one's own products versus a competitor's products, or paying cash for buyback versus offering a store credit. One interviewee (1) has engaged policymakers on a state-by-state level to address these issues in order to implement their buyback and resale model in local markets.

In the normative context (Appendix B, Table B4 of Supporting Information S1), interviewees referred to institutional work directed at connecting individuals with organizational values, changing traditional meanings, and creating new norms and networks. One interviewee (6) highlighted the challenging of existing norms around the American cultural aversion to rental as opposed to buying and owning furniture. They characterized rental as incompatible with the “American dream,” particularly for older generations who may see rental business as predatory, even equating it with “rent-to-own,” while younger generations (Millennials and Gen Z) are more open to renting.

As for cultural-cognitive institutional work (Appendix B, Table B5 of Supporting Information S1), we observed substantial evidence of isomorphic mimicry, with multiple interviewees (1 and 7) mentioning other businesses coming to them in order to learn what is working and how they can recreate those successes themselves. One interviewee (2) noted that the uncluttered simplicity of their showroom is itself a means of communicating the value of owning less to potential customers: “part of the reason why we're here is to help you have less stuff, because your life will be better.”

#### Disrupting institutions

We observed little evidence of true disruption of existing institutions, although a number of interviewees referred to what they were doing as disruptive.

Some interviewees (1 and 6) did offer examples of attempting normative disruption in terms of undermining existing assumptions and beliefs. Interviewee 1, for example, characterized their work as challenging the dominant linear economic model embedded in consumer psychology, saying that “it's a really strong psychology to try and disrupt.”

In the cultural-cognitive context, companies mentioned they tried to remove important transaction costs, for example, to encourage circular behavior. For example, a novel logistics system pursued by Fernish was seen as disruptive in the transition to CBMs that require solid reverse logistics. While for incumbent retailers product returns are “their worst nightmare,” for Fernish, it is “expected for every product” and it is their “business model” enabled through their customized logistics system (interviewee 17). In the case of Philips, the trade-in model was seen as something that could be disruptive through providing the customer with a “one-stop shop” for the circular economy (interviewee 5), making it an easy solution for them to responsibly deal with a product, allowing for it to be refurbished or recycled at the moment of trade-in (interviewee 4). This model was driven by its “Close the Loop Pledge” and Philips’ broader sustainability ambitions (interviewee 5).

Finally, in terms of regulation, companies mentioned examples of educating governmental actors about the circular economy and trying to remove unnecessary regulatory barriers (interviews 1 and 7). For example, interviewee 1 mentioned legislation that prohibits specific ″buyback resale structures based on the other types of buyback resell structures. It was important to find ways to make their buyback model viable: “So we did have to change legislation, which is quite a bit disruptive, and also helps for other retailers to come into the space in those areas if they were a little gunshot before working with regulators” (Interviewee 1). Interviewee 7 mentioned that he went to “testify (…) in front of the U.S. Trade Commission on the impact of remanufacturing and the restrictions placed on us by different companies.” This company is an example of a “gap exploiter” (Den Hollander & Bakker, 2016) remanufacturing other Original Equipment Manufacturer's (OEMs'). furniture, exploiting a gap in the market not (yet) captured by the OEMs themselves.

By interacting with governmental actors in this way, such companies could pave the way for CBMs for other companies.

## DISCUSSION

### Circular business models in a US context

This study contributes novel insight on how companies institutionalize CBMs in a given context by bridging theory on institutional work on how organizations influence dominant institutions, and ecologies of business models, on how business models develop vis-à-vis other business models. To investigate the institutionalization of CBMs, we investigated how CBMs emerge in the US context.

First, we found evidence for a variety of business models, including circular design for durability (including a premium model); PSS (rental and lease); and buy or take back with direct resale or remanufacturing. This goes far beyond earlier research identifying only a limited number of innovative CBMs in the S&P 500 context—the largest companies listed on US stock exchanges (Ritala et al., 2018).

Second, we found that, perhaps unsurprisingly, the US context is large and diverse, which has several implications. First, the size of the country coupled with a limited national rail transportation network increases the “low-carbon logistics challenge” to support a sustainable circular economy (e.g., reverse logistics). This echoes earlier work stating that geography may inhibit or drive the success of CBMs (Han et al., 2022). Second, the US’ size means plentiful landfill space without “not in my backyard” (NIMBY) risk, which may be more of a problem in Europe. Furthermore, landfilling is still relatively cheap in many places as mentioned by some of the interviewees. Third, and related to this, doing business in the United States means that there are a variety of environmental policies in “sustainability hotspots” like California or New York, but a blanket circular economy policy like that of Europe is missing (Ghisellini et al., 2016). Even some of the lower “R” strategies are not yet solidified, and regulation compelling companies to recycle is lacking. The National Framework for Advancing the US Recycling System (launched in 2019) is a start (EPA, 2022b), but it is not yet widely embedded in business operations.

Yet, some successful national-level policies are emerging. While a recent report from RMI (2022) concluded that the Inflation Reduction Act (IRA) would lead to 37 million additional EVs on US roads by 2032, the IRA has also effectively incentivized recycling of EV batteries, resulting in a rush to construct battery recycling facilities (Carey et al., 2023; ICCT, 2023). These incentives effectively make battery recycling profitable by allowing manufacturers using recycled battery materials to claim them as US made—even if the materials originated abroad—thus qualifying them for IRA subsidies. Some interviewees mentioned the importance of the IRA in driving sustainable innovation. Implementing these kinds of policies across other sectors could help to create a business case for circularity and overcome some state-level gaps. Indeed, recent research from Moktadir et al. (2020) confirms that legislation is one of a handful of critical success factors for driving circular economy practices.

Third, the institutional context in the United States is quite different from Europe (often the basis for comparison by interviewees) and not yet very conducive to CBM innovations. Many of the interviewees mentioned the United States is behind the EU on circular policy, which makes it harder to make a case for CBMs. Business cases are focused on *cost reductions* (e.g., secondhand or remanufacturing), *aspirational products* (e.g., vintage or durable), or *convenience* (e.g., one-stop shop for sustainability and product as a service). This adds to understanding of business cases for sustainable business models, with evidence on real business cases (Schaltegger et al., 2012). However, there are tensions between cheap disposal costs and lack of company buy-in to potential value of reuse of materials and products versus global supply chain instabilities and the need for access to critical rare earth elements.

Finally, dominant consumer attitudes in the United States make the circular economy implementation challenging. Although younger generations seem more open to new business models, interviewees noted that a lot of CBMs like secondhand or rental do not fit “the American dream.” This requires a lot of “educating” in a business context, at industry or sector fora or conferences, through advertising, by speaking with legislators (see also Bocken & Shirahada, 2025). Several interviewees referred to a “combined” approach where multiple forms of institutional work come together.

### Pathways to CBM implementation in the US context

**FIGURE 1:**
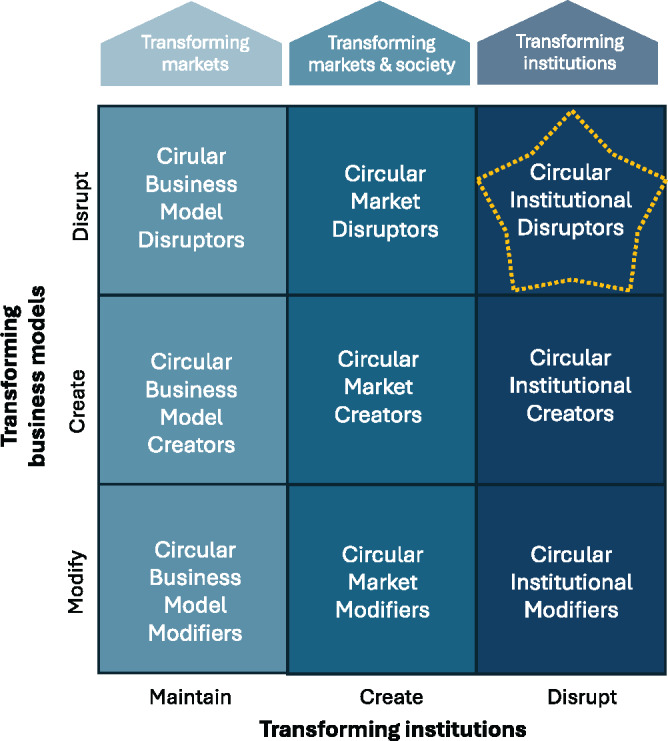
Pathways for circular business model innovators. *Source*: This study, building on Scott (2013), Lawrence and Suddaby (2006), Boons and Bocken (2018). *Note: Maintaining institutions was out of scope for the present study*.

A country with institutional voids like the United States brings both challenges and opportunities in the circular economy transition as companies need to create their own pathways without clear institutional support. The interviewees in this study innovate via new CBMs in support of the wider circular economy transition. First, we found that companies sought to modify, create, and disrupt business models, experiencing both mutualistic/symbiotic dependencies and competing/parasitic dependencies during this process validating strategies in Boons and Bocken (2018). Second, we found that most companies seek to help create new institutions, and to a lesser extent, disrupt them. The focus on creating rather than disrupting is slightly different from findings in the sharing economy where companies seem to take both paths equally (see Zvolska et al., 2019). Conceptually, the possible pathways for transforming business models and institutions may be framed as in Figure 1. While the ultimate goal might be to disrupt both institutions and business models, labeled here “Circular Institutional Disruptors,” in reality most of the businesses were operating in the space of creating institutions while modifying, creating, or seeking to disrupt business models, aiming to transform market and societal dynamics toward circularity (middle column, Figure 1). Changes at the full institutional level also including policy (final column; transforming institutions) was less prominent in our study, although there were some successes: for example, remanufacturing has been promoted with legislators as *job creation*, providing an additional “business case” for legislators beyond resource savings.

### Circular Business Institutionalization Framework

Institutional work has helped make institutional theory more dynamic and applicable by increasing visibility of the practices and activities companies might adopt to maintain, change, and disrupt institutions (e.g., Lawrence & Suddaby, 2006; Zvolska et al., 2019). The “ecologies of business models” theory (Boons & Bocken, 2018) adds a dynamic perspective on how CBMs co-evolve and has been further operationalized into a framework to help companies understand their positioning (Bocken et al., 2019).

We found value in both the ecologies of business models perspective and institutional work in understanding how new CBMs evolve in the United States The frameworks revealed evidence of new business models emerging, disrupting, competing, and co-existing with other business models. They also revealed the extent to which company actions were disruptive and how these relate to changing or disrupting the normative and cultural-cognitive contexts and regulatory landscape. We observed that internal cultural-cognitive work can play an important role in driving the development and adoption of CBMs, which confirms earlier research suggesting that such work can ultimately contribute to a firm's transition toward circularity (Sancak, 2023). We also found overlaps and synergies in the sense that both concepts might be used to not only *explain* but also potentially *plan* the move to a CBM. Applying these concepts can reveal dependencies on other business models and actors, as well as relationships with existing dominant institutions.

**FIGURE 2:**
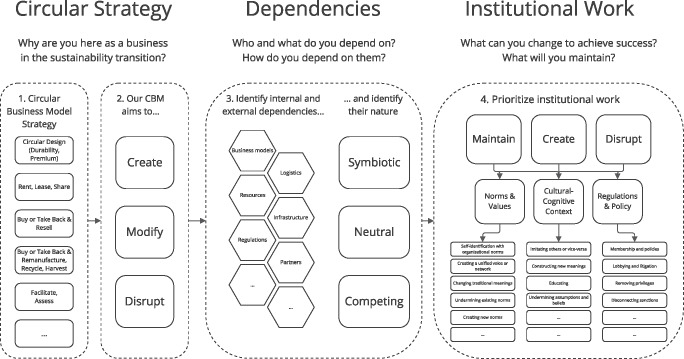
Circular Business Institutionalization Framework. Building on Bocken and Shirahada (2025), Boons and Bocken (2018), Bocken et al. (2019), Lawrence and Suddaby (2006), and Zvolska et al. (2019).

To this end, we bring these concepts together in a unifying framework (Figure 2 based on Table 1), the *Circular Business Institutionalization Framework*, to help businesses institutionalize CBMs. First, it is important to identify the type of business model in question and clarify its purpose, as well as whether it aims to create, modify, or disrupt business models in the existing industry. So, in the first step, we include the different types of CBMs we identified in this study as a starting point. Second, it is essential to be aware of the dependencies that exist and whether they are symbiotic, neutral, or competing (see Boons & Bocken, 2018). For example, we found that the secondhand business model at IKEA competes with valuable retail space of the existing new products. Davies Office has been exploiting gaps in the market by remanufacturing Original Equipment Manufacturer (OEM)'s furniture (Den Hollander & Bakker, 2016). Although this initially led to a “love–hate relationship,” OEMs are now “embracing them.” Dependencies are evolving but awareness of these and what one could do about them is essential. The final step involves institutional work for a CBM to become successful. In our study, there were ample examples of companies trying to (1) shift norms and values (not only with customers but also within the company and industry), (2) transform the cultural-cognitive context (attending conferences and educating others, e.g., about remanufacturing), and (3) conduct regulatory institutional work by understanding potential legislative barriers and then trying to remove or work around them.

The *Circular Business Institutionalization Framework* may guide future research in the area of circular business (models). It could help generate awareness for companies on their context of influence, and support policy makers in developing policy frameworks that institutionalize the circular economy. It can also serve an important role in helping firms develop strategies for implementing CBMs, as there is currently little guidance in this area grounded in both theory and empirical research (Puglieri et al., 2022)

### Implications for management

We found a variety of CBMs being pursued by companies including: (1) Premium pricing and durability, (2) PSS like rental and lease, (3) Buy- or take-back and resale models, (4) Buy- or take-back and reprocessing models, and (5) Facilitator and assessment models. Such CBMs co-exist with so-called linear business models. Yet, companies have successfully identified relevant value propositions and target markets for new CBMs with business cases focused on aspirational products, convenience, and cost reductions. We found that for CBMs to be successful, institutional work is often necessary to influence norms and values (e.g., buying quality over quantity), what is culturally acceptable (e.g., secondhand), and how regulation may be of benefit or interpreted for the CBM to be successful (e.g., what is considered “waste”). Awareness of the institutional context and how to influence this appears essential. Managers may use the Pathways for CBM innovators and Circular Business Institutionalization Framework to position their CBM vis-à-vis collaborators and competitors, and develop institutional leverage points for their organization.

### Limitations and future research

The main limitation of the work relates to the sampling. Through snowballing a full population of businesses may rarely be reached. While all companies achieved scale, the sample included a heterogeneous set of companies, some founded in the United States while others were not. There are a number of fruitful areas which future research could explore as revealed during our exploratory study.

First, future research could examine the EU–US context, including existing and future regulatory spillover from the EU to the United States (Bocken et al., 2025). Many interviewees pointed to contextual differences between the two, highlighting the challenges in developing a business case for a CBM in the United States compared to the EU. Meanwhile, a number of emergent EU regulations connected to the circular economy will impact US companies doing business in Europe, but it is unclear to what extent US companies are aware of or prepared for these.

Second, additional research is needed regarding how CBMs can develop and operationalize reverse logistics systems. This is especially true in the United States, where regulations vary by state and can create substantial reverse logistics challenges (i.e., for buy-back models), but it is arguably an underexplored challenge across geographies, including in the EU.

Finally, more research is needed as it relates to consumer attitudes in the United States and how those attitudes might delimit the potential success of CBMs. Put another way, research could investigate to what extent companies wishing to implement CBMs may need to actively work to reshape consumer attitudes around disposability, new versus used, etc. In the US context, it could be interesting to explore to what extent consumer attitudes are geographically correlated with those states which have introduced stronger circular economy policies.

## CONCLUSIONS

This study investigated the institutionalization of CBMs. Circular economy is not yet institutionalized in the United States. Because of its unique characteristics including these institutional voids, and being home to some of the world's largest companies, the United States is an important and relevant context to investigate. Through interviews with several companies, we investigated the following question: How do CBMs emerge in the US context?

As for types, we found evidence of CBMs focused on circular design, PSS (rental, lease, etc.), product take- or buy-back followed by remanufacturing, refurbishment, or small repairs and resale. We identified companies who were transforming their linear business models, gap exploiters filling gaps in the market, as well as companies positioning entirely new scaled CBMs successfully into the market or those facilitating others through platforms or evaluation of CBMs.

As for emergence, we found companies that were creating entirely new models, disrupting the market, or modifying existing models. There were several dependencies on existing business models and infrastructures—including sometimes a pure dependency on OEM stock by gap exploiters—and otherwise a sometimes challenging co-existence with existing infrastructure. Although many companies did not see themselves as too disruptive, we found examples of companies both creating and disrupting institutions at a normative, cultural-cognitive, and regulatory levels.

Finally, based on the theoretical lenses we took—ecologies of business models and institutional work—we developed Pathways for CBM innovators and a Circular Business Institutionalization Framework that could both guide future business actions, policy-making, and academic research.

## Supplementary Information

**Supporting Information S1**: This supporting information provides interview questions and summary interviewee responses.

## Data Availability

Research data are not shared.
