# Supplementary material for: Institutionalization of circular business models in the United States
Source: J Ind Ecol. 2025 Nov 8;29(6):2353–66. doi: 10.1111/jiec.70115 (PMC13279581; doi:10.1111/jiec.70115)
Supplement: Supplementary file 1 — Supporting Information S1: This supporting information provides interview questions and summary interviewee responses. [file 44498_2025_2906029_MOESM1_ESM.docx]

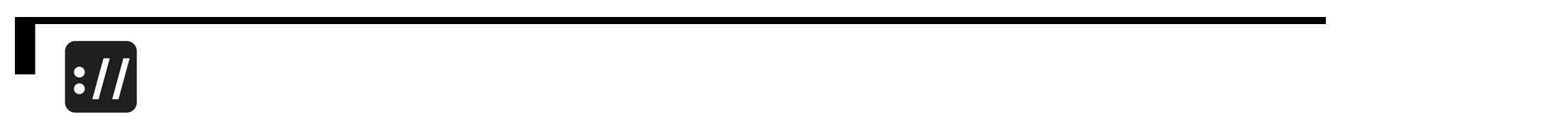


SUPPORTING INFORMATION FOR:

Bocken, N., Coffay, M. Institutionalizing circular business models in the U.S. *Journal of Industrial Ecology.*


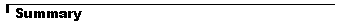


This supporting information provides interview questions and summary interviewee responses.


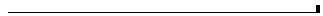


*Tables in the supporting information with more than 50 numerical data points (e.g., data tables with 5 columns and 10 rows) should be provided in a numerical format (e.g., comma-separated value files or a spreadsheet). For a template for spreadsheets used as supporting information, please visit* <http://jie.click/templates>*.*

**Appendix A. Interview questions for semi-structured interviews**

[Introduction]

1. What is your role at *Company …*?

[Company’s own business model]

1. What is the circular economy vision/ aim of your company*?*
2. Can you describe *your circular business model*? What new and unique offering does it bring?

[Business model in relation to others (ecologies of business models, Boons & Bocken, 2018)]

1. Would you mainly describe your business model as aiming to modify, or disrupt the existing dominant linear business model in your main industry, or mainly positioning a new one?

- *Modifying:* improve the existing model, minimize, or reduce the dependency on less sustainable/ linear business models
- *Disrupt*: destroy the linear business model, e.g. by outcompeting them on their key products or services
- *Create*: maximize the positive contribution to circular business models (e.g. creating a new more favourable model, creating a favourable institutional infrastructure like norms, practices, and rules)

1. To what extent does the business model depend on others and how?
   - Does your circular business model depend for its *success* on the *presence of* *others*? [dependency]
   - Do *other* business models benefit from *your* presence? [mutualism and symbiosis]
   - To what extent does the success of your business models depend on the *failure* of another? [competition]
   - To what extent does your business model compete for *limited resources or space?* [competition]

[institutional work, Zvolska et al., 2019]

1. Normative context:
   - Is your business model changing or aiming to change, drive or establish new norms? [creating]
   - Is your company undermining moral grounds of ‘linear economy’ consumption lifestyles, behaviour or purchasing? [disrupting]
   - To what extent is your company trying to create new identities, that may also appeal to other businesses? (e.g., Company X business model as the driver of the sustainable mobility transition) [creating]
   - To what extent is your company undermining the moral grounds or identity of linear business models? [disrupting]
   - Is your company involved in any networks to create a united voice or identity or new codes of conduct? [creating]
2. Regulatory context:
   - Is your business involved in any lobbying/ shaping policies and regulations? [creating]
   - To what extent is your company involved in lobbying and litigation to *remove privileges*, preferential treatment, subsidies, or rewards from established or competing practices, technologies or actors? [disrupting]
   - Is your business involved in setting new standards (e.g., labels) or organisational forms (e.g., benefit corporation) [creating]
3. Cultural-cognitive context:
   - Is your business model leveraging the association with existing taken for granted practices, technologies etc to improve acceptance and long-term survival? [creating]
   - Did you imitate any business models, or did others try to imitate your business model? [creating]
   - Are you trying to create new constructs (e.g., artefacts, symbols, concepts) that help build legitimacy for the circular economy? [creating]
   - Are you educating other organizations in the field of the circular economy? [creating]
   - Is your company removing important transaction costs (e.g., Airbnb’s platform enabled home sharing) or undermining established cognitive assumptions (Airbnb made home sharing safe through peer-to-peer feedback and the sharing platform). [disrupting]

[other factors and closing]

1. Is your company involved in any other important circular economy related practices not mentioned before or influenced strongly by other factors, or organizations not mentioned before in the field of circular economy?
2. Do you have any questions for me?

**Appendix B. Interviewee responses**

**Table B1. Ecologies of business models: positioning of circular business model**

|  | **Interview quotes** |
| --- | --- |
| Modifying the model/ dependencies | “I think it's a bit of a modification. I don't think it's disrupting anything, because I think other businesses have sort of started to (...) sell you something that lasts longer. . We are very good at that. I mean, every product we sell can be repaired or fixed, or (..) is just going to last [but] We don't have a buyback program. I think it's something that we would like to do. It comes up a lot, but we encourage people to sell their stuff privately if they don't need it anymore, because there's a healthy market for it (interviewee 2)  “I don't necessarily see it as disruptive for the industry. I think it's actually really hard to do sustainability. Well in the US. To be very honest with you. There is a lack of national regulation and a patchwork of solutions across the geography of the United States that makes it hard.” (interviewee 18) |
| Disrupting the linear model | “No one else in the US are doing this, at least not doing it very successfully if they are. So it is a disruptive service, and that we are taking back our own product and paying customers for it. and it's not a return policy, because it's not 30, 60, 93, 365 days. It's even if the product is 15 years old, we'll buy it back.” (interviewee 1)  “we'll give you the same warranty and performance and execution, and save you money, and do the right thing for the environment. And so they viewed us as disruptive to your point, because they did not like the fact that we were in what they consider to be their marketplace”. (interviewee 7)  “I definitely think it's a little disruptive because we do as I said, both recycling and resell. So we really feel like we're kind of the best of both worlds” (interviewee 10)  “I mean, I don't know that we're trying to be outwardly disruptive, I think, were trying to be internally disruptive and trying to be internally disruptive and challenge the linear take- make-waste ideaI ... I mean within my team particularly, I think I challenge everyone to to sort of think about things in a creative way, and to find companies that have the same ethos as us and want to push the envelope” (interviewee 13) |
| Creating an entirely new circular model | “You know. I I'm proud to say that we we really we really were the first, I say, Next Gen Furniture rental player” (interviewee 6)  “we have (...) an amazing software to efficiently manage the business that if you're launching out of the gates. (..) that's just not gonna optimize your operations (..) Everyone else would say, Oh, it's like, it's return and reverse and circular logistics. But we're like, no (...) we fundamentally built that into our (..) business model. So logistics for us would look crazy for [a large retailer] because they're expecting nothing to come back. And when something actually does come back. It's a huge problem for retailers. They don't know what to do with it. It's like their worst nightmare - returns are everyone's worst nightmare. For us, like returns are expected for every product" (interviewee 17) |

**Table B2. Ecologies of business models: dependencies**

|  | **Interview quotes** |
| --- | --- |
| Mutualistic and symbiotic dependencies | “We've decided as a company that we cannot do this alone. That partnership is the only way we're going to have a meaningful impact. So we acknowledged that partnership. Partnering is very important part of this model.” (Interviewee 1) |
|  | “I think it is clear that sustainability is a team sport. No one company or organization can do it alone. So we have an open ecosystem approach, where we can engage with partners and even competitors via open APIs which can be used to exchange data (…) The challenge is that circularity is still not a well-defined space so there are many overlapping solutions, and the trick is finding the right partners with limited overlap in order to create the most shared value for our customers.” (Interviewee 14) |
| Competing or parasitic dependencies | “other furniture startups emerged on the scene (..) some have come and gone, which, which you know again, wasn't, wasn't a big surprise to us, because, really, in order for this business model to work, you have to offer furniture that's gonna last long and more than a few turns and can be restored can be refinished can be brought back to new” (interviewee 6)  “So our sector was not embraced by the OEMs. They constantly tried to throw obstacles in our way. Performance, optical, safety issues where we constantly had to prove we want the same level playing field (Interviewee 7).  “when the articles come back into the store they need to have some place to sit until we're ready to put them back on the floor, even though there is a really quick turnaround from coming back to recovery getting, and then getting resold. I believe it's something around 72 hours that it's with us. It's a quick turnaround, but it's still some place to sit, whether it's waiting to be that (sic) brought back to a saleable state or on the showroom floor, and the more that our as-is/circular hub takes up room in the store, that's less room for other [new] things to sell.” (Interviewee 1) |

**Table B3. Examples of creating institutions in the regulatory context**

| **Creating institutions (regulatory context)** | |
| --- | --- |
|  | **Interview quotes** |
| Lobbying and litigation, advocacy | “So what we've had to do in some States is work with policy and policymakers and regulatory agencies to get the legal support to be able to do this. There are some languages in some States that prohibit some buy back resale structures based on the other types of buy back resell structures. So we've worked with them for them to understand that we're only buying back our own product. We're not paying cash for it. People are getting a store credit for it. So it's not us paying cash for a good that may be, may have some kind of theft history on it, or so it's just our goods that we're getting store credit for. So we did have to change legislation, which is quite a bit disruptive, and also helps for other retailers to come into the space in those areas if they were a little gun shy before about working with regulators.” (Interviewee 1)    "So ISRI is our National Lobbyist group. And I'm the chair of the Electronics Division for ISRI, currently. So I'm working on some things. You know, just getting our company name out there and in the spotlight. And as a result of that, yeah, we get asked for partnerships and different things all the time, which is great. In addition to that we've got a really nice strategic relationship with one of the largest scrap companies or landfill companies in the world believe it or not" (Interviewee 10) |
| Delimiting organizational fields e.g., through membership and policies | “We formed a group back in 2,003, called the Paper Working group. It was composed of companies like [large well-known business X, Y, and Z] and a range of other companies that all purchase large amounts of paper, and we're trying to place requirements on mills and on forest products companies. And and we were all. We were all asking for sort of the same.. the same ships, but we were all sending out our own questionnaires and trying to get information, and we came to the reality that you know what we were doing was actually creating more confusion in the marketplace because there was not harmonization right of of what the market wanted. And as a result, you know the data that came back from any of the companies we we didn't know whether we were comparing, you know, say, apples, apples, and apples or apples, oranges and banners. So so, as a result, you know, we we found that those kinds of collaboratives were very, very important.” (Interviewee 8) |

**Table B4. Examples of creating institutions in the normative context**

| **Creating institutions (normative context)** | |
| --- | --- |
|  | **Interview quotes** |
| Self-identification (connecting individual with organizational values) | “One, you think about the employee base. And I think so much of what we're doing, being that it's a mission driven business, tied to the circular economy and really elevating furniture as a category into the circular economy, that really does unify our team” (Interviewee 17) |
| Changing traditional meanings | “… in the US is it not.. It’s frowned upon. It's not the first - second hand is not the first choice new. And you know what's, what's hot now is where people in the US go first. So there is a push, this undercurrent that we're feeling that we're trying to to get rolling around, making second hand the first choice.” (Interviewee 1) |
| Creating new norms | “Our team is very excited about changing consumer behavior in that direction [circularity], because it's just better for the world. And that's kind of one element. We have an operating value around care for the communal, and that's, you know, all relative stakeholders into business, which you know includes the planet” (Interviewee 17)  “The other aspect to is that, you know, consumers still are a little bit sheepish about renting their furniture. There's something about like being able… Find your home, buying your furniture, you know. There's something about like making it. The American dream and rental…are not keen, for, you know, older populations. It's it's still largely looked down upon because I think of the rental center atmosphere. But for Millennials and Gen. Z. You know, they they're very keen on it. It's just getting the word out.” (Interviewee 6) |
| Organizing for a unified voice, creative normative networks | “There are a couple of organizations and associations that we belong to with other retailers where it's a safe space for us to talk about this. So we're definitely trying to collaborate. And I guess, collaborate to figure out what are the pain points? What are the successes we can build on? What have other coalitions, organizations, or associations done that have been successful, that we can model and use that same format to have a successful resale/second hand market or structure or partnership to build from? So there's definitely a desire to have that. But it's not in existence yet.” (Interviewee 1) |

**Table B5. Examples of creating institutions in the cultural-cognitive context**

| **Creating institutions (cultural-cognitive context)** | |
| --- | --- |
|  | **Interview quotes** |
| Isomorphic mimicry, imitation | “So it is a little disruptive in the retail sense of it. And it is also in the disruption is that other retailers would like to emulate it (interviewee 1)  “Other groups are coming to us and say, can you explain what's or how your model works” (interviewee 7)  “I'm always a big believer that that businesses react to to models, even if they're pilots that are already built and are working right? And you can some data. I think I think to be the first in anything many times is difficult. But then you can find an analogous example, or a corresponding kind of a model that you can use to explain to people why you believe, even though this isn't the same product” (interviewee 8) |
| Constructing new meaning systems | “Getting people to realize that that's part of the reason why we're here is to help you have less stuff, because your life will be better. We know this for fact, and we can see it. When people walk into our stores, our stores are very clean. They're very sparse, and they have a few things. And people say, oh, I wish I lived here. And I think you could live here if you've got rid of your stuff.” (Interviewee 2) |
| Educating | “Again around our trials and tribulations and getting this getting it propped up. And also what the value proposition is for the consumer. So I think there is some education happening. We've been asked to talk about it at several round tables or panel discussions. So there's definitely interest from other retailers about how we're doing it, and how well it's going.” (Interviewee 1)  I think at this point I've had no circularity discussion with the US. For all of my colleagues there that I've told, ‘I'm starting circular economy’, not one of them have understood what it is. Most of them believe it's either I'm starting a recycling business…or we’re starting their leasing business…So, the understanding of circularity is quite low. I think that the understanding of carbon neutrality is quite high.” (Interviewee 15) |
